# Importance of measuring testosterone in enzyme-inhibited plasma for oral testosterone undecanoate androgen replacement therapy clinical trials

**DOI:** 10.4155/fso.15.55

**Published:** 2015-11-01

**Authors:** Sylvain Lachance, Om Dhingra, James Bernstein, Stéphanie Gagnon, Caroline Savard, Nathalie Pelletier, Nadine Boudreau, Ann Lévesque

**Affiliations:** 1 inVentiv Health clinical, Québec, Canada; 2 SOV Therapeutics, Morrisville, NC, USA

**Keywords:** enzyme inhibitor, hydrolysis, method validation, testosterone, testosterone undecanoate

## Abstract

**Aim:**

Testosterone undecanoate (TU) is metabolized by nonspecific esterases in blood to testosterone (T). Typical clinical practice has been to analyze testosterone in human serum. The degradation of TU to testosterone was evaluated in conditions typically used in clinical studies.

**Methods & Results:**

Freshly collected whole blood was fortified with TU at known concentration. Serum was prepared and T concentration was determined by LC–MS/MS. It was observed that TU degrades extensively to T in human blood under conditions typical of harvesting serum causing overestimation of T concentration of up to 243%. These results were confirmed in a clinical study in which serum and plasma samples were compared.

**Conclusion:**

It was demonstrated that T must be analyzed in enzyme-inhibited plasma when TU is the administered medication.

## Abstract (lay abstract)

Testosterone undecanoate (TU) is metabolized into testosterone. Its degradation in whole blood into testosterone was studied in conditions typically used in clinical trials. It was observed that TU degrades extensively to testosterone in human blood under conditions typical of harvesting serum, causing overestimation of testosterone concentration. It was demonstrated that testosterone must be analyzed in enzyme-inhibited plasma when TU is the administered medication. Using serum for testosterone quantitation in a clinical trial for androgen replacement therapy would bias the conclusions on formulations adjustment and use.

**Figure 1.**
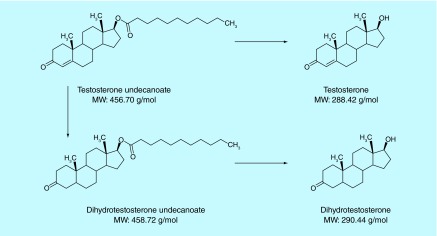
**Molecular structures of testosterone undecanoate, dihydrotestosterone undecanoate, testosterone and dihydrotestosterone.**

**Figure 2.**
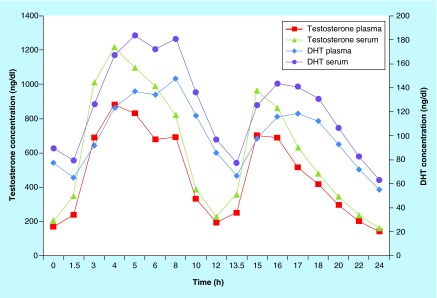
**Testosterone and dihydrotestosterone NaF/Na_2_ ethylenediaminetetraacetate plasma versus serum mean concentrations.** DHT: Dihydrotestosterone.

Testosterone undecanoate (TU), a prodrug of testosterone (T) is metabolized by nonspecific esterases in blood to testosterone [1,2]. More specifically, TU is metabolized partly in the intestinal wall into 5-alpha-dihydrotestosterone undecanoate (DHTU). In blood and tissues, TU is hydrolyzed to testosterone and DHTU to dihydrotestosterone (DHT) (Figure 1). Two intramuscular (IM) and one oral testosterone replacement therapy (TRT) products using TU have been approved in the USA (IM; Aveed^®^), Europe (oral; Andriol^®^ or Restandol^®^ Testocaps™; IM, Nebido^®^), Canada (oral; Andriol^®^) and many other countries.

Typical practice in the bioanalytical industry has been to analyze T and DHT in human serum [3–7]. To minimize conversion of TU to testosterone during the preparation of serum from blood, published literature in preclinical studies of TU has reported the use of potassium fluoride in sample collection tubes [8]. However, published clinical data on TU have used standard serum tubes without an esterase inhibitor [2–7]. One publication on the validation of a bioanalytical method for testosterone analysis concluded that TU is stable in blood [9]. Other methods developed to measure testosterone in plasma samples were published during the last decades [10,11]; however, even if these methods gave acceptable performance, they were not developed using enzymatic inhibitors and did not avoid the potential conversion of the TU and DHTU into testosterone and DHT.

As part of the development and validation procedure for a bioanalytical method, the stability of the analyte in the sample should be demonstrated from the blood drawn into a collection tube through the separation of the plasma/serum from the red blood cells and other blood components. In the case of TU, we have to demonstrate that the ester does not undergo hydrolysis into T during sample processing. The TU concentration is sometimes more than 20- to 30-times higher than the testosterone concentration in serum or plasma [12], and therefore even minimal degradation of TU impacts the measured concentration of T.

During investigation on the stability of TU in whole blood as a sample matrix, it was observed that TU converts rapidly into T. The rate of conversion was rapid and was dependent on the TU concentration. Experiments were performed to demonstrate the instability of TU and its impact on T concentration. The potential conversion of the metabolite dihydrotestosterone undecanoate (DHTU) to DHT is also discussed. Data are also presented on the best choice of sample matrix and collection tube for clinical measurement of T when TU is administered. The impact of the TU conversion on the T concentration could lead to inappropriate decisions on the starting dose and dose adjustments for patients on TU containing TRT products.

## Materials & methods

Four validated methods were used for the present work for the collection and processing of samples: testosterone and DHT in serum tubes, testosterone and DHT in sodium fluoride/potassium oxalate (NaF/K_2_C_2_O_4_) plasma tubes, testosterone and DHT in sodium fluoride/sodium ethylenediaminetetraacetate (NaF/Na_2_ EDTA) plasma tubes and TU and DHTU in NaF/Na_2_ EDTA plasma tubes.

Briefly, T and DHT were extracted from the sample matrix (serum or plasma) by liquid-liquid extraction. 0.500 ml of the deuterated internal standards working solution and the buffer solution (Trizma Base 500 mM, Sigma, Oakville, Canada) is added to 0.300 ml of matrix. After adequate mixing, 5 ml of extraction solvent methyl *tert*-butyl ether (MTBE from EMD, Toronto, Canada) was added, mixed well for 15 min and centrifuged at 1950*g* for 5 min at room temperature. The top layer was then transferred to a borosilicate tube and evaporated to dryness. The dry residue is reconstituted with 0.150 ml of mobile phase and injected onto the HPLC column (ACE Excel 2 C18-PFP, 100 × 3 mm, 2 µm from Life Science, Canada) using reverse phase chromatography conditions. The mobile phase is a mixture of methanol (EMD, Canada), Milli-Q Type water and acetic acid (EMD, Canada) in a proportion of 70/30/0.2. Detection was done by MS (LC–MS/MS) using the AB SCIEX API 5000 (Toronto, Canada). Positive ionization modes using the TurboIonSpray were optimized with the mass transitions 290.4→97.0 amu for testosterone, 291.3→255.4 amu for DHT, 294.4→97.0 amu for testosterone-d_5_ and 294.4→258.4 amu for DHT-d_3_.

TU and DHTU were extracted from 0.150 ml of matrix by an automated liquid–liquid extraction using 1.5 ml of methyl *tert*-butyl ether and 0.200 ml of ammonium formate pH 5 buffer (Fluka, Oakville, Canada). Deuterated internal standards were used for both analytes. The analytes are extracted, the upper phase transferred and evaporated to dryness. The dry residue was reconstituted with the mobile phase (Milli-Q water/methanol 10/90 ammonium formate 5 mM, formic acid 0.1%), injected onto the HPLC column (Waters BEH Phenyl, 50 × 3 mm, 1.7 µm, Canada) and analyzed by LC–MS/MS using the AB SCIEX API 5000. Positive ionization modes using the TurboIonSpray were optimized with the mass transitions 457.4→271.3 amu for TU, 476.5→273.3 amu for DHTU, 462.5→276.3 amu for TU-d_5_ and 497.6→273.3 amu for DHTU-d_21_.

### Method validation for the determination of testosterone, DHT, TU & DHTU

The methods were validated as per the most recent US FDA and EMA validation guidelines [13,14]. During the method validation, the accuracy, precision, within-run, between-run, selectivity, matrix effect as well as the stability (stability in whole blood at 4°C, short-term stability in matrix at room temperature, freeze–thaw stability at -20°C/-80°C, long-term stability at -20°C/-80°C) were evaluated.

For T and DHT in serum, the method was validated according to the guidelines. However, stability of the analytes in whole blood in the presence of TU and DHTU did not meet the acceptance criteria due to apparent degradation of TU and DHTU into T and DHT, respectively.

### Evaluation of the conversion of TU in whole blood

During the method development and validation, the conversion of TU into testosterone or secondarily DHTU to DHT in whole blood was verified. In fresh whole blood, a quantity of TU at different concentrations was added to the matrix. These tests were performed in presence or absence of additives. If no additives were used, the aliquots were set-aside for at least 30 min (minimal period required for complete coagulation) at room temperature before processing to serum. The plasma or serum was separated by centrifugation and testosterone and DHT concentration was analyzed.

### Application of the selected assay to a clinical study

A clinical study was performed at the inVentiv Health Clinical facilities where fifteen asymptomatic hypogonadal male subjects aged 18 to 75 years were administered an oral dose of TU twice daily for 84 consecutive days under fed conditions. Each volunteer was informed on the risk of procedure and risk of the clinical study by signing the informed consent. On day 84, serum and plasma samples were collected predose and at 1.5, 3, 4, 5, 6, 8, 10, 12, 13.5, 15, 16, 17, 18, 20, 22 and 24 h after the morning dose. One of the main objectives of this study part was to compare the levels of T and DHT measured with a validated method in NaF/Na_2_ EDTA plasma to the levels measured in serum.

Pharmacokinetic (PK) parameters were assessed using samples collected on day 84: Ln-transformed area under the concentration–time curve (AUC_0–24_) and the maximum concentration of analyte (C_max0–24_) obtained with the study samples collected from 0–24 h after the morning dose of TU on last study day 84 were used to compare the levels of testosterone, DHT, TU and DHTU measured in serum in comparison to the levels measured in plasma.

## Experiments & results

### Evaluation of the conversion of TU in whole blood without additives over time

TU at a concentration of 160,000 ng/dl and DHTU at a concentration of 74,000 ng/dl were added to freshly collected whole blood containing no additive and mixed. Aliquots were set aside for 30, 90 and 240 min at room temperature and then the serum was separated by centrifugation and analyzed.

A significant increase of the T and DHT concentrations in the samples was observed suggesting instability of TU and DHTU in the whole blood samples. Testosterone and DHT concentrations increased from 1816.9 to 6172.8 ng/dl, from 176.6 to 1012.9 ng/dl, respectively, when TU and DHTU was incubated in whole blood without additive from 30 to 240 min at room temperature (Supplementary Figure 1). It must be noted that no testosterone was added to the samples.

These results demonstrated that TU and DHTU degrade into T and DHT during the clotting period of the sample collection and serum harvesting. To improve the accuracy of T and DHT measurement, inhibition of the enzymatic degradation appears necessary.

### Impact of the TU concentration on T in whole blood during clotting with time

Fresh whole blood was collected without anticoagulant from a single donor and the endogenous T level was determined as 23.75 ng/dl. The whole blood sample was fortified with different concentrations of TU, ranging from 1500 to 70,000 ng/dl and set aside for clotting for 30 and 60 min. Different TU concentrations were tested in order to evaluate the potential impact of varying TU levels on T concentration during a clinical study in which TU will be administered. The clotting time were set to mimic the incubation times typically used during a clinical trial. The resulting serum was analyzed for T and compared with the endogenous level of T.

Results are presented in Table 1. The concentrations of T increased over time and depended on the concentration of TU used to fortify the samples, showing that TU degrades rapidly into T in whole blood during clotting and serum harvesting. Moreover, even low concentrations of TU have an impact on testosterone concentrations.

### Comparison of different additives to prevent the conversion of TU to T

Since the stability of blood samples containing TU is hypothesized to be influenced by the activity of esterases in the blood, sample collection was performed with different collection tubes (Table 2) to investigate the optimum sample collection tubes.

Fresh whole blood from one donor (subject 2) was collected in each type of tube and endogenous T level was determined. Serum tubes were allowed to clot for 30 min at room temperature, plasma tubes were set aside for 10 min at 4°C. The plasma or serum was obtained by centrifugation and analyzed for T. Results are presented in the Table 2. It was observed that NaF may introduce a negative bias in the testosterone measurement as the degree of difference increases with the percent of NaF in the tubes. However, for whole blood collected with tubes with NaF (0.43%), this bias was less significant (-0.7%). It is noted that the incubation temperature for this tube is room temperature as serum is to be harvested, contrary to plasma for other tubes with NaF. As it is explained further in another section, partitioning of the analyte is suspected to be impacted by NaF. This partitioning is likely sensitive to temperature resulting in different bias at room temperature than 4°C.

The same collection tubes were used to evaluate the degradation of TU. Fresh whole blood from one donor (subject 1) was collected in the different collection tubes. Each sample was fortified with TU at a final concentration of 60,000 ng/dl. This concentration was evaluated as the potential C_max_ of TU. This recommendation is based on the ratio relationship between DHT and T levels as well as TU and DHTU levels [7]. For each tube, a comparison sample and a stability sample were prepared. For the serum tubes, one aliquot was allowed to clot for 30 min (comparison samples), the other for 60 min at room temperature (stability samples). For the RST tubes, the time was set at 10 (comparison samples) and 40 min (stability samples) since the clotting is very fast. NaF 0.43% tubes and RST (rapid serum tubes) were processed as the serum. For the plasma samples (NaF/Na_2_ EDTA, NaF/K_2_C_2_O_4_, EDTA K_2_ and BD^TM^ P800 containing Protease and Esterase Inhibitor tubes) one aliquot was set aside for 10 min at 4°C (comparison samples) and another for 60 min at 4°C (stability samples). After the respective duration, the plasma or serum was obtained by centrifugation and analyzed for testosterone and compared. The condition of each sample and results are also presented in the Table 2. It is clear from the results that temperature has an effect on the conversion as demonstrated by the samples that were collected at room temperature. In addition, esterase inhibition with NaF does not completely prevent hydrolysis of TU to T, but minimizes it when used along with additives (EDTA or K_2_C_2_O_4_). Considering this, three types of anticoagulants were selected for further investigation: K_2_ EDTA, NaF/Na_2_ EDTA and NaF/K_2_C_2_O_4_. It was found that EDTA alone did not prevent conversion of TU to T, and that NaF/K_2_C_2_O_4_ and NaF/Na_2_ EDTA were equivalent (see Supplementary Tables 1 & 2).

### Comparison of testosterone levels in serum of different donors in plasma with NaF/Na_2_ EDTA & NaF/K_2_C_2_O_4_

Since testosterone is usually analyzed in the serum in the biomedical laboratories, it is preferable to identify anticoagulants that can give endogenous T concentration in plasma equivalent to the serum. A comparison of the endogenous level of testosterone for eight different donors was done in serum, NaF/Na_2_ EDTA plasma and NaF/K_2_C_2_O_4_ plasma. As the method was intended for clinical trials applications, eight different donors were tested. Results are presented in Table 3. The mean percent of difference of endogenous T concentrations between serum and NaF/Na_2_ EDTA plasma was 11.7 and 13.8% between NaF/K_2_C_2_O_4_ plasma and serum. The difference in endogenous T concentrations measured with NaF/Na_2_ EDTA and NaF/K_2_C_2_O_4_ tubes is not clinically significant, although there is a slight preference for NaF/Na_2_ EDTA due to the slightly lower bias.

Wang *et al*. suggested that the use of NaF introduces a negative bias of 20% compared with serum samples [9]. A negative bias is consistent with our investigation and a mean% bias of -11.7% (Table 3) was observed for the testosterone basal level in NaF/Na_2_ EDTA plasma compared with human serum in normal blood donors. As the addition of NaF is important to minimize the hydrolysis of TU, this negative but reproducible bias is not clinically significant when compared with the uncontrolled clinically meaningful increase (i.e., about 30–35%) of testosterone concentration in the presence of TU as would occur in serum samples.

### Determination of endogenous level of testosterone in whole blood (NaF/K_2_C_2_O_4_, NaF/Na_2_ EDTA, BD™ P800 containing protease & esterase inhibitor)

It was observed in the above experiments that NaF (refer to Table 2) suppressed the testosterone concentration. In order to determine if this bias is related to ion suppression or testosterone degradation in presence of NaF, freshly collected whole blood in different tubes were analyzed. Here, tubes with additives were used to analyze testosterone in whole blood. As it was observed previously that there was no difference in endogenous level of testosterone in serum versus K_2_ EDTA plasma, this tube was used as a comparator. Serum and plasma of the same samples were harvested and analyzed for testosterone concentrations.

Testosterone concentrations in whole blood were compared for each anticoagulant and the results showed no significant difference between the anticoagulant tested (Supplementary Tables 3 & 4). The harvested serum or plasma was analyzed for testosterone and the negative bias due to NaF was again observed. As testosterone concentration in whole blood is similar between each anticoagulant, it suggests that NaF does not cause ion suppression to the LC–MS/MS detection. It could also suggest that partitioning between erythrocytes and plasma is slightly different when using tubes with or without NaF in the tube.

In addition, quality controls were prepared in human serum and were analyzed with a calibration curve prepared in NaF/K_2_C_2_O_4_ plasma. The mean percent of bias of the quality controls in human serum were all within 2% of the nominal concentrations, showing that the sodium fluoride does not affect the quantitation of testosterone. Results are provided in Supplementary Table 5.

### Validation in human NaF/Na_2_ EDTA & NaF/K_2_C_2_O_4_ plasma

Complete validations were done for plasma samples obtained from NaF/K_2_C_2_O_4_ or NaF/Na_2_ EDTA collection tubes in accordance with the most recent FDA and EMA validation guidelines [13,14]. All tests met the acceptance criteria. The equivalence between the anticoagulants NaF/K_2_C_2_O_4_ and NaF-Na_2_ EDTA was confirmed.

The assay for the determination of T and DHT in human plasma or in human serum was validated over the dynamic range of 100–30,000 and 50–5000 pg/ml for T and DHT, respectively. Linearity was demonstrated with coefficient of determination (r^2^) higher than 0.9927 for both analytes in all batches. Accuracy (% bias) and precision (coefficient of variation) were within 6.7 and 8.5%, respectively. The extraction recovery was also determined and was higher than 85 and 83% for T and DHT, respectively.

TU and DHTU method was validated in human plasma over the dynamic range of 1–1000 and 0.5–500 ng/ml for TU and DHTU, respectively. The coefficient of determination (r^2^) was higher than 0.9935 for TU and DHTU in all validation batches. Accuracy and precision were demonstrated with% bias within 1% and coefficient of variation within 10%. The extraction recovery was of 81 and 84% for TU and DHTU, respectively.

Additional tests were done during the method validation to prove the stability of the analytes and that no other conversion occurred (e.g., glucuronide degradation) (Supplementary Tables 6 & 7). Testosterone glucuronide had no impact on the quantitation of T and DHT when low and high T and DHT level quality controls fortified with testosterone glucuronide were submitted to stress conditions such as freeze–thaw cycles, incubation at room temperature, 4°C, long-term storage at -20 or -80°C. The impact of the glucuronide was also assessed in whole blood during sample collection and handling.

Moreover, the possible degradation of TU and DHTU into T or DHT was assessed when low and high T and DHT level quality controls were submitted to freeze–thaw cycles, incubation at room temperature or 4°C and long-term storage at -20 and -80°C. No increase of the T and DHT concentrations were observed during these stability studies. Since the degradation was specifically observed in whole blood during sample collection, the impact of TU and DHTU was also assessed in in low- and high-quality controls prepared in whole blood and set aside for 110 min at 4°C. The results obtained during validation clearly show that the use of plasma NaF/Na_2_ EDTA is adequate for quantitation of T and DHT.

However, for the method in human serum, the evaluation of the degradation of TU and DHTU during sample collection in whole blood did not meet the acceptance criteria. Indeed, an increase of the T concentration of up to 83% was observed after 60 min at room temperature of clotting time compared with the regular 30 min while DHT concentration increased by 140%.

### Comparison of the TU to T conversion depending on the TU spiking solvent used

Wang *et al*. [9] published that addition of TU to blood did not increase the measured serum T levels even at high concentrations, which is not in accordance to the findings presented here. We decided to investigate further to better understand the discordance between the conclusions. Literature descriptions of sample preparations include a stock solution prepared in ethanol and diluted in phosphate buffer saline (PBS) [9]. In the experiments carried out in the study reported here, it was observed that the stock and spiking solution preparation is very important. A stock solution of TU was prepared in ethanol and diluted with PBS prior to being added to whole blood. Results from this procedure were compared with those typically used in our experiments with TU stock prepared and diluted using methanol, prior to being added to whole blood. It was observed that a precipitate occurred in the PBS solution after a freeze–thaw cycle.

To investigate this effect further, freshly collected whole blood was collected in conventional serum tubes and in NaF/K_2_C_2_O_4_ tubes. Each whole blood tube was fortified with TU at a final concentration of 60,000 ng/dl with solution prepared either in methanol or in ethanol/PBS; each experiment was performed in triplicate. Serum samples were incubated at room temperature for 30 and 60 min, and plasma samples were incubated for 10, 30 and 60 min at 4°C. T and TU were analyzed in the plasma and serum samples obtained.

Table 4 summarizes the results obtained. Testosterone and TU concentrations are stable when the stock and working solution of TU was prepared in methanol and plasma obtained from the whole blood sample. In contrast, the T and TU concentrations in serum samples prepared using either methanol or ethanol/PBS spiking solutions were variable. The TU concentrations for serum samples spiked using TU in methanol appears stable, but the T concentrations are not, presumably because of the absence of an enzyme inhibitor (such as sodium fluoride). We also note that while the T concentrations observed in plasma samples after spiking with TU from ethanol/PBS are stable, they are markedly lower than T concentrations in plasma samples spiked with TU from methanol.

More consistent results were obtained using the combination of TU prepared and spiked using methanol solutions, and measured from plasma samples. The temperature of sample preparation also appears important to obtain accurate results.

### Comparison of concentrations in serum & plasma in a clinical study

The experiments described thus far involved limited numbers of sources of samples, and thus require confirmation in a larger study. Data reported in Table 5 were obtained from a cohort of 15 subjects undergoing a clinical study of a novel formulation of TU. Table 5 presents summaries of the comparison of the PK results for T and DHT obtained using serum and plasma samples for each analyte. Figure 2 presents the mean analyte concentrations obtained from the serum and plasma samples.

For testosterone, results obtained from serum and plasma cannot be considered equivalent since the AUC_0–24_ and C_max0–24_ values are outside of the 80–125% standard equivalence acceptance range for the 90% CI of the ratio (serum/plasma); the ratio was 130.02% for the AUC_0–24_ and 134.01% for the C_max0–24_. For DHT, although the 90% CI for the ratio (serum/plasma) for the AUC_0–24_ fall within the 80–125% acceptance range (122.40%), serum and plasma cannot be considered equivalent due to significant difference between C_max0–24_ values (130.61%).

## Conclusion

During the method development of the assay for the determination of testosterone and DHT, it was observed that the ester prodrug TU and its metabolite DHTU degrade extensively to testosterone and DHT in whole blood.

When serum is harvested in the absence of enzyme inhibitors, the hydrolysis of TU and DHTU to T and DHT occurs during whole blood collection and processing to serum. Multiple experiments demonstrated that serum is not the preferred matrix for the analysis of T and DHT when TU is the administered medication.

During the experiments, it was demonstrated that:

The conversion of TU to testosterone is extensive and continues over time in whole blood when no enzyme inhibitors are present;
Even low concentrations of TU have an impact on measured testosterone concentrations;
Temperature of collection influences the conversion rate;
The addition of an esterase inhibitor, namely NaF, is important to minimize the hydrolysis of TU to T.

The results of the validation of the methods in plasma NaF/K_2_C_2_O_4_ and NaF/Na_2_ EDTA for T, DHT, TU and DHTU demonstrate that the assay is accurate, precise and that the analytes are stable under the conditions tested. Moreover, stability testings in presence of TU show that hydrolysis is minimized with NaF/K_2_C_2_O_4_ or NaF/Na_2_ EDTA, accompanied by low temperature sample collection.

A comparison of T and TU concentrations for serum and plasma samples spiked using either ethanol stock solutions of TU diluted into PBS or methanol stock solutions of TU diluted with methanol showed that consistent and high recoveries were only obtained using the methanol solutions and plasma samples. Whether this is a solubility or partitioning phenomena remains unclear. However, the use of PBS for the dilution of TU solution may introduce a bias in the evaluation of the impact of TU degradation on the T exposure.

Comparison of T and DHT concentrations obtained from serum and plasma samples for a clinical study demonstrates that the conversion of TU to testosterone and DHTU to DHT occurred when serum samples were collected. The observed conversion was proportional to the concentrations of TU and DHTU. The PK parameters for plasma and serum testosterone concentrations were not bioequivalent in terms of C_max0–24_ and AUC_0–24_. For DHT, the C_max0–24_ values were also outside the bioequivalence range. Results from these analyses found the day 84 AUC_0–24_ were 29.97% higher when measured in human serum versus plasma, in a clinical study of 15 hypogonadal men administered TU twice daily for 12 weeks.

Although NaF/K_2_C_2_O_4_ gave excellent performance in terms of preventing the hydrolysis of TU, the investigation suggests that NaF/Na_2_ EDTA is the anticoagulant of choice since the bias in testosterone level with the conventional serum tubes is slightly lower than with the NaF/K_2_C_2_O_4_ matrix.

The observed instability of TU in serum samples would appear to render a bioanalytical method for testosterone based on serum sample analysis inappropriate due to the inaccuracy of the results obtained. It would significantly bias the decisions on the starting dose and dose adjustments during TRT as these decisions will be based on the T concentration that will be overestimated in serum. It is concluded that the determination of testosterone and DHT should be performed in human plasma samples obtained using NaF/Na_2_ EDTA collection tubes.

## Future perspective

The determination of testosterone has been performed in serum for many years. However, for TRT, specifically when esters of testosterone are used as prodrug, the analysis of testosterone in serum causes overestimation of testosterone exposure. This investigation demonstrates that analysis in plasma combined with an enzyme inhibitor is to be used for future bioanalytical studies.

**Table 1.** **Conversion of testosterone undecanoate into testosterone according to the testosterone undecanoate concentrations and the incubation time.**

**TU Concentration Fortified (ng/dl)**	**Duration of incubation (min)**	**Concentration testosterone measured (ng/dl)**	**% difference vs TU = 0 (%)**	**% difference 30 vs 60 min (%)**
0	0	23.75	–	–
1500	30	36.76	54.8	24.6
	60	45.81	92.9	
10,000	30	152.66	542.8	65.8
	60	253.18	966.0	
30,000	30	306.02	1188.5	73.9
	60	532.19	2140.8	
70,000	30	732.49	2984.2	72.4
	60	1262.81	5217.1	

TU: Testosterone undecanoate.

**Table 2.** **Conversion of testosterone undecanoate into testosterone in whole blood collected with different collection tubes and endogenous level of testosterone in different collection tubes.**

**Matrix**	**Anticoagulant**	**Comparison sample^†^**	**Testosterone concentration (ng/dl)**						
			**Subject 1**					**Subject 2**	
			**Stability sample**	**Comparison sample**	**% Change vs unfortified serum**	**Stability sample**	**% change**	**Endogenous level^‡^**	**% change vs unfortified serum**
Serum	Without anticoagulant not fortified	30 min at RT	NA	370.61	Reference	NA	NA	287.21	Reference
Serum	Without anticoagulant	30 min at RT	60 min at RT	1270.19	242.7	1967.80	54.9	282.51	-1.6
Serum	Rapid serum tube without anticoagulant	10 min at RT	40 min at RT	814.41	119.7	1228.29	50.8	247.77	-13.7
Serum	NaF 0.43%	30 min at RT	60 min at RT	992.38	167.8	1592.15	60.4	285.29	-0.7
Plasma	EDTA K_2_	10 min at 4°C	60 min at 4°C	441.91	19.2	528.99	19.7	262.77	-8.5
Plasma	NaF (0.15%)-Na_2_ EDTA	10 min at 4°C	60 min at 4°C	392.77	6.0	452.53	15.2	247.89	-13.7
Plasma	NaF (0.25%)-K_2_C_2_O_4_	10 min at 4°C	60 min at 4°C	409.15	10.4	461.07	12.7	233.78	-18.6
Plasma	BD™ P800 containing protease and esterase inhibitor	10 min at 4°C	60 min at 4°C	479.57	29.4	720.30	50.2	283.29	-1.4

Samples were fortified with 60,000 ng/dl of testosterone undecanoate.

^†^Samples are incubated for 30 min when blood is collected to allow the coagulation and to mimic the clinical sample handling of serum samples and for plasma samples, whole blood is incubated for 10 min at 4°C as handled in clinic.

^‡^Endogenous level was determined in matrices from a different donor than the testosterone undecanoate conversion tests.

EDTA: Ethylenediaminetetraacetate; NA: Not applicable; RT: Room temperature.

**Table 3.** **Comparison of testosterone endogenous levels between serum and plasma with NaF/Na_2_ ethylenediaminetetraacetate and NaF/K_2_C_2_O_2_ in different donors.**

**Donors**	**Testosterone concentration in serum (ng/dl)**	**Testosterone concentration in NaF/Na_2_ EDTA (ng/dl)**	**% change vs serum**	**Testosterone concentration in NaF/K_2_C_2_O_4_ (ng/dl)**	**% change vs serum**
1	301.62	273.63	-9.3	254.22	-15.7
2	309.23	273.18	-11.7	280.06	-9.4
3	479.70	424.16	-11.6	399.71	-16.7
4	555.39	485.15	-12.7	457.20	-17.7
5	511.13	467.45	-8.6	507.68	-0.7
6	576.13	487.55	-15.4	483.81	-16.0
7	490.22	432.20	-11.8	421.20	-14.1
8	263.80	230.30	-12.7	211.26	-19.9
**Mean**	435.90	384.20	-11.7	376.89	-13.8
**SD (±)**	124.31	106.86		112.97	
**CV (%)**	28.5	27.8		30.0	

EDTA: Ethylenediaminetetraacetate.

**Table 4.** **Testosterone and phosphate buffer saline concentrations in serum or plasma harvested from blood spiked with testosterone undecanoate working solution prepared in phosphate buffer saline or methanol; spiking level 60,000 ng/dl.**

**Experiments**	**Endogenous level in serum**	**Endogenous level in plasma**	**30 min serum RT**	**60 min serum RT**	**Plasma 10 min 4°C**	**Plasma 30 min 4°C**	**Plasma 60 min 4°C**
**Mean testosterone concentration (ng/dl)**							
Whole blood spiked with TU in PBS	255.4	221.7	295.4	349.0	236.0	232.9	231.1
Whole blood spiked with TU in methanol	255.4	221.7	854.8	1255.4	322.5	339.9	320.5
**Mean TU Concentration (ng/dl)**							
Whole blood spiked with TU in PBS	N/A	N/A	3991.0	5892.3	74408.7	54088.0	26618.7
Whole blood spiked with TU in methanol	N/A	N/A	97110.3	96132.3	97235.0	95628.0	93816.3

PBS: Phosphate buffer saline; RT: Room temperature; TU: Testosterone undecanoate.

**Table 5.** **Pharmacokinetic results obtained from serum and NaF-Na_2_ ethylenediaminetetraacetate plasma.**

**Analyte**	**Parameter**	**Ratio (%)**	**90% geometric CI (%)**		**Intra-subject CV (%)**
			**Lower**	**Upper**	
Testosterone	AUC_0–24_	130.02	128.06	132.01	2.34
	C_max0–24_	134.01	128.66	139.58	6.29
Dihydrotestosterone	AUC_0–24_	122.40	120.33	124.50	2.63
	C_max0–24_	130.61	125.38	136.05	6.30

**Executive summary**

It was demonstrated that testosterone must be determined in plasma with sodium fluoride as enzyme inhibitor when the esters of testosterone are used as prodrug.
Testosterone undecanoate degrades extensively into testosterone in whole blood at room temperature and without enzyme inhibitor, when serum is to be harvested.
This degradation is time and temperature dependent.
This was confirmed with incurred samples when serum was compared with plasma.
Testosterone is to be determined in NaF/Na_2_ ethylenediaminetetraacetate plasma and kept at low temperature to avoid overestimation of concentration.
