# Supplementary material for: Importance of measuring testosterone in enzyme-inhibited plasma for oral testosterone undecanoate androgen replacement therapy clinical trials
Source: Future Sci OA. 2015 Nov 1;1(4):FSO55. doi: 10.4155/fso.15.55 (PMC5137954; doi:10.4155/fso.15.55)
Supplement: Supplementary file 1 [file fso-01-55-s1.doc]

**Supplemental Tables**

**Suplemental Figure 1. Conversion of Testosterone Undecanoate and Dihydrotestosterone Undecanoate to Testosterone and Dihydrotestosterone Over Time in Whole Blood at Room Temperature**


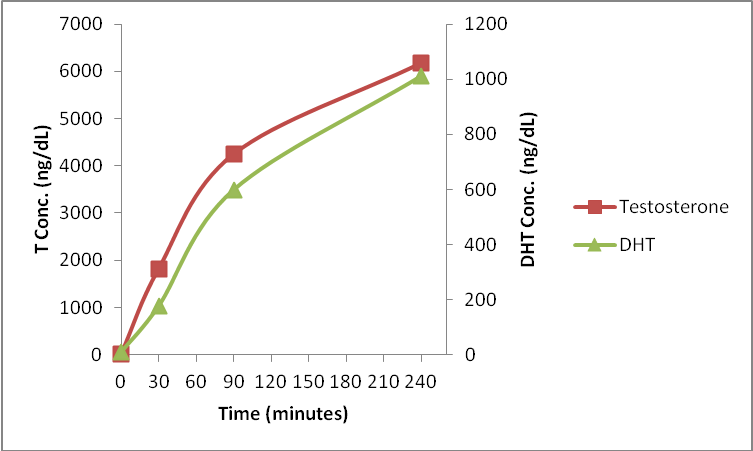


Endogenous level: 14.5 ng/dL of Testosterone and 8.9 ng/dL of DHT

Samples were fortified with 160000 ng/dL of TU and 74000 ng/dL of DHTU.

**Suplemental Table 1. Conversion of TU into Testosterone in Plasma from Do**nors With Different Anticoagulant Over Time

| **Anticoagulant** | **Duration** | **Testosterone Concentration (ng∕dL)** | | | | | | | |
| --- | --- | --- | --- | --- | --- | --- | --- | --- | --- |
|  |  | **Donor 1** | **% Change vs. 10min** | **Donor 2** | **% Change vs. 10min** | **Donor 3** | **% Change vs. 10min** | **Donor 4** | **% Change vs. 10min** |
| **EDTA K_2_** | 10 min 4°C | 628.27 | N/Ap | 624.21 | N/Ap | 564.43 | N/Ap | 473.27 | N/Ap |
|  | 30 min 4°C | 648.74 | 3.3 | 667.84 | 7 | 530.39 | -6 | 512.34 | 8.3 |
|  | 60 min 4°C | 686.53 | 9.3 | 808.07 | 29.5 | 606.62 | 7.5 | 569.21 | 20.3 |
|  | 60 min RT | 1172.24 | 86.6 | 1823.61 | 192.1 | 1441.05 | 155.3 | 1273.69 | 169.1 |
|  |  |  |  |  |  |  |  |  |  |
| **NaF-Na_2_EDTA** | 10 min 4°C | Not done | N/Ap | 414.69 | N/Ap | 385.50 | N/Ap | 364.49 | N/Ap |
|  | 30 min 4°C |  | N/Ap | 425.92 | 2.7 | 397.22 | 3 | 375.61 | 3.1 |
|  | 60 min 4°C |  | N/Ap | 460.18 | 11 | 424.61 | 10.1 | 394.43 | 8.2 |
|  | 60 min RT | Not done | | | | | | |  |
|  |  |  |  |  |  |  |  |  |  |
| **NaF-K_2_C_2_O_4_** | 10 min 4°C | 490.05 | N/Ap | 405.16 | N/Ap | 408.56 | N/Ap | 378.99 | N/Ap |
|  | 30 min 4°C | 538.41 | 9.9 | 423.26 | 4.5 | 425.52 | 4.2 | 386.49 | 2 |
|  | 60 min 4°C | 503.92 | 2.8 | 479.48 | 18.3 | 497.82 | 21.8 | 446.41 | 17.8 |
|  | 60 min RT | 804.67 | 64.2 | 991.42 | 144.7 | 812.15 | 98.8 | 809.41 | 113.6 |

Samples were fortified with testosterone undecanoate at a concentration of 60000 ng/dL

**Suplemental Table**2. Conversion of TU into Testosterone (ng/dL) Over Time for Different Donors in Plasma

| **Donors** | **Testosterone Concentration (ng/dL)** | | | | | |
| --- | --- | --- | --- | --- | --- | --- |
|  | **NaF/Na_2_EDTA** | | | **NaF/K_2_C_2_O_4_** | | |
|  | **10 min 4°C** | **60 min 4°C** | **% Change** | **10 min 4°C** | **60 min 4°C** | **% Change** |
| **1** | 305.15 | 350.36 | 14.8 | 382.99 | 382.31 | -0.2 |
| **2** | 332.13 | 356.03 | 7.2 | 379.23 | 375.45 | -1 |
| **3** | 514.06 | 502.56 | -2.2 | 537.83 | 529.77 | -1.5 |
| **4** | 557.11 | 553.41 | -0.7 | 596.48 | 540.31 | -9.4 |
| **5** | 560.83 | 557.76 | -0.5 | 556.58 | 604.46 | 8.6 |
| **6** | 526.60 | 559.14 | 6.2 | 608.17 | 609.94 | 0.3 |
| **7** | 562.12 | 510.66 | -9.2 | 664.52 | 526.08 | -20.8 |
| **8** | 283.66 | 313.38 | 10.5 | 374.61 | 343.71 | -8.2 |
| **Mean** | 455.21 | 462.91 | 3.3 | 512.55 | 489.00 | -4.0 |
| **SD (±)** | 124.57 | 104.71 |  | 116.77 | 106.24 |  |
| **CV (%)** | 27.4 | 22.6 |  | 22.8 | 21.7 |  |

**Suplemental Table 3. Testosterone Concentration in Whole Blood using Different Collection Tubes**

|  | **Testosterone Concentrations in Whole Blood (ng/dL)** | | | |
| --- | --- | --- | --- | --- |
|  | **EDTA K_2_** | **NaF/Na_2_EDTA** | **NaF/K_2_C_2_O_4_** | **P800** |
|  | 19.58 | 17.52 | 18.32 | 18.51 |
|  | 18.65 | 18.43 | 18.10 | 18.72 |
|  | 18.33 | 18.82 | 17.77 | 19.17 |
| **Mean (ng/dL)** | 18.85 | 18.27 | 18.06 | 18.79 |
| **SD (±)** | 0.65 | 0.67 | 0.27 | 0.34 |
| **CV%** | 3.4 | 3.7 | 1.5 | 1.8 |
| **% Change  vs. EDTA K_2_** | N/Ap | -3.1 | -4.2 | -0.3 |

*****Each sample was analyzed in triplicate

**Suplemental Table 4. Testosterone Concentration in Serum or Plasma**

|  | **Testosterone Concentrations in Plasma (ng/dL)** | | | | |
| --- | --- | --- | --- | --- | --- |
|  | **Serum** | **EDTA K_2_** | **NaF/Na_2_EDTA** | **NaF/K_2_C_2_O_4_** | **P800** |
|  | **31.69** | 30.92 | 27.89 | 26.81 | 32.10 |
|  | **32.02** | 31.65 | 28.82 | 27.02 | 32.42 |
|  | **32.91** | 31.38 | 29.07 | 27.34 | 32.81 |
| **Mean (ng/dL)** | **32.21** | 31.32 | 28.59 | 27.06 | 32.44 |
| **SD (±)** | **0.63** | 0.37 | 0.61 | 0.26 | 0.36 |
| **CV%** | **1.9** | 1.2 | 2.1 | 1.0 | 1.1 |
| **% Change vs. Serum** | **N/Ap** | -2.8 | -11.2 | -16.0 | 0.7 |

*****Each sample was analyzed in triplicate

**Suplemental Table 5. Accuracy of Quality Controls in Human Serum with a Calibration Curve in Human NaF/K_2_C_2_O_4_**

|  | **Low Serum QC (10.0 ng/dL)** | | **High Serum QC (1000.0 ng/dL)** | |
| --- | --- | --- | --- | --- |
|  | **Testosterone Conc. Found (ng/dL)** | **% Bias** | **Testosterone Conc. Found (ng/dL)** | **% Bias** |
|  | 9.95 | -0.6 | 987.12 | -1.3 |
|  | 9.88 | -1.2 | 971.41 | -2.9 |
|  | 9.59 | -4.1 | 979.24 | -2.1 |
|  | 10.16 | 1.6 | 976.50 | -2.4 |
|  | 9.92 | -0.8 | 989.17 | -1.1 |
|  | 9.89 | -1.1 | 979.83 | -2.0 |
| **Mean** | 9.90 | -1.0 | 980.54 | -2.0 |
| **SD (±)** | 0.19 |  | 6.63 |  |
| **CV%** | 1.8 |  | 0.7 |  |

**Suplemental Table 6. Impact of TU, DHTU, Testosterone Glucuronide and DHT Glucuronide on the Testosterone Concentrations Under Different Stability Conditions in NaF/Na_2_**EDTA Plasma

| **Stabilities** | **% Bias** | |
| --- | --- | --- |
|  | **Low QC (40.0 ng/dL)** | **High QC (2300.0 ng/dL)** |
| Freeze/Thaw at -20°C (4 Cycles) | -5.8 | -3.3 |
| Freeze/Thaw at -80°C (4 Cycles) | -5.8 | -3.4 |
| Short-Term at RmT for 23 hrs | -2.5 | 0.2 |
| Short-Term at 4°C for 21 hrs | -4.2 | 0.6 |
| Long-Term at -20°C for 14 days | -1.9 | -1.8 |
| Long-Term at -80°C for 14 days | 2.1 | -3.1 |
| Whole Blood on ice for 110 min | -11.1 | 8.3 |
| Post-Preparative at RT for 69 hrs | 14.0 | 10.1 |

Low QC samples were fortified with testosterone undecanoate (3000 ng/dL), dihydrotestosterone undecanoate (1500 ng/dL), testosterone glucuronide (800 ng/dL) and dihydrotestosterone undecanoate (400 ng/dL). High QC samples were fortified with testosterone undecanoate (219000 ng/dL), dihydrotestosterone undecanoate (235100 ng/dL), testosterone glucuronide (93600 ng/dL) and dihydrotestosterone undecanoate (21900 ng/dL). The%Bias must be within 15%.

**Suplemental Table 7. Impact of TU, DHTU, Testosterone Glucuronide and DHT Glucuronide on the Dihydrotestosterone Concentrations Under Different Stability Conditions in NaF/Na_2_**EDTA Plasma

| **Stabilities** | **% Bias** | |
| --- | --- | --- |
|  | **Low QC (19.2 ng/dL)** | **High QC (430.0 ng/dL)** |
| Freeze/Thaw at -20°C (4 Cycles) | -14.6 | -10.2 |
| Freeze/Thaw at -80°C (4 Cycles) | -14.9 | -10.7 |
| Short-Term at RT for 23 hrs | -6.1 | 2.5 |
| Short-Term at 4°C for 21 hrs | -9.1 | -0.5 |
| Long-Term at -20°C for 14 days | -8.7 | -4.4 |
| Long-Term at -80°C for 14 days | -8.1 | -5.4 |
| Whole Blood on Ice for 110 min | -6.1 | 0.3 |
| Post-Preparative at RT for 69 hrs | 5.1 | 8.6 |

Low QC samples were fortified with testosterone undecanoate (3000 ng/dL), dihydrotestosterone undecanoate (1500 ng/dL), testosterone glucuronide (800 ng/dL) and dihydrotestosterone undecanoate (400 ng/dL). High QC samples were fortified with testosterone undecanoate (219000 ng/dL), dihydrotestosterone undecanoate (235100 ng/dL), testosterone glucuronide (93600 ng/dL) and dihydrotestosterone undecanoate (21900 ng/dL). The%Bias must be within 15%.
